# Autorização para uso *off-label* pode ser benéfica para o Sistema Único de Saúde?

**DOI:** 10.1590/0102-311XPT032623

**Published:** 2023-06-26

**Authors:** Marisa da Silva Santos, Márcia Gisele Santos da Costa, Bernardo Rangel Tura, Priscila Torres, Sandro José Martins, Fotini Santos Toscas

**Affiliations:** 1 Instituto Nacional de Cardiologia, Rio de Janeiro, Brasil.; 2 Conselho Nacional de Saúde, Brasília, Brasil.; 3 Biored Brasil, Brasília, Brasil.; 4 Hospital Universitário de Brasília, Brasília, Brasil.; 5 Instituto de Saúde, Secretaria de Estado da Saúde de São Paulo, São Paulo, Brasil.

Com relação ao artigo publicado na seção *Perspectivas* de CSP ^1^, gostaríamos de apresentar uma visão diferente de um grupo de pesquisadores envolvidos de modo direto no processo de incorporação da Comissão Nacional de Incorporação de Tecnologias no Sistema Único de Saúde (Conitec) desde a criação da Comissão de Incorporação de Tecnologias do Ministério da Saúde (Citec).

A crítica à possibilidade de incorporação de medicamentos sem registro em bula (*off-label*), embora seja um ponto de vista válido, retirou a discussão do contexto. A decisão foi tomada em um cenário de maior interesse do sistema público de saúde e contra interesses da indústria farmacêutica.

Como apontado por Reinaldo Guimarães, pesquisador do Núcleo de Bioética e de Ética Aplicada da Universidade Federal do Rio de Janeiro (UFRJ) em entrevista ao portal Outra Saúde ^2^, “*...o uso off-label pelo SUS pode ser benéfica, principalmente quando há estratégias comerciais da indústria em fazer combinações para que o registro de um medicamento não seja realizado para determinada indicação para não prejudicar outra indústria que já tenha outro medicamento - bem mais caro - para aquela indicação no mercado público...*”.

O contexto inicial da discussão sobre medicamentos *off-label* na Conitec foi a degeneração macular relacionada à idade (DMRI), em que o bevacizumabe apresentava maior eficiência que o ranibizumabe. A DMRI é a causa mais comum de cegueira em idosos em países desenvolvidos. A prevalência da DMRI no Brasil é estimada em cerca de 10% em indivíduos com mais de 80 anos ^3^. A comparação entre o medicamento de referência (ranibizumabe) e o incorporado (bevacizumabe) foi feita em vários ensaios clínicos randomizados e revisões sistemáticas de alta qualidade, como a de Solomon et al. ^4^, na qual vários desfechos (ganho de acuidade em um e dois anos de acompanhamento, mudança média na acuidade, qualidade de vida e redução na espessura da retina) não apresentaram diferença entre o medicamento registrado e o *off-label*. A única diferença foi o custo - o tratamento anual com bevacizumabe pode representar uma redução de cerca de R$ 20.000,00 por olho tratado (cálculo com base no Sistema Integrado de Administração de Serviços Gerais - SIASG). Manter a proibição de uso *off-label* em casos excepcionais como o descrito é do interesse apenas da indústria farmacêutica e pode impedir o acesso de um tratamento eficaz para muitos pacientes.

A Agência Nacional de Vigilância Sanitária (Anvisa), embora provocada pela Conitec, não tem se posicionado a favor de incluir em bula indicações comprovadamente eficazes, apenas por contrariar interesses econômicos, alegando que “*não é da sua competência*” ^5^, conforme processo nº 25000.056762/2020-43, disponível no Sistema Eletrônico de Informações do Ministério da Saúde (SEI/MS).

Outros cenários de destaque em que o uso *off-label* representa o melhor interesse público são: imunossupressores para transplante cardíaco, hepático e pulmonar, inclusive everolimo e tacrolimo, alvos de política das parcerias de desenvolvimento produtivo (PDP) com o Instituto de Tecnologia em Fármacos, Fundação Oswaldo Cruz (Farmanguinhos/Fiocruz) ^6^. Produzir medicamentos no Brasil reduz a dependência externa, e a aprovação do uso *off-label*, desde que embasada em evidências adequadas, burla práticas predatórias do mercado.

Um dos casos de *off-label* que mais gerou mobilização social foi o do micofenolato de mofetila nos protocolos clínicos e diretrizes terapêuticas (PCDT) de lúpus eritematoso sistêmico, que apresenta evidências da atuação da Conitec com a avaliação de tecnologias em saúde (ATS) pautada em necessidades sociais. O medicamento tinha comprovações científicas robustas e elementos suficientes para que a Anvisa pudesse atualizar sua indicação em bula. A Conitec declarou que, apesar das fortes evidências, por questões regulatórias, o micofenolato de mofetila não foi incorporado no PCDT de lúpus em 2017 e foi incluído somente em 2022, cinco anos depois. Nesse período de cinco anos, potencialmente, muitos pacientes com lúpus perderam seus rins por falta de acesso.

Outro ponto de discordância com as opiniões dos autores é a respeito da criação de três comitês em substituição ao plenário, com a inclusão de representantes dos Núcleos de Avaliação de Tecnologias em Saúde (NATS). A mudança é adequada e resulta de demandas do próprio plenário, que muitas vezes necessita apoio de um metodologista no suporte à tomada de decisão. A existência de comitês diversos, em especial para equipamentos, permitirá melhorias no processo de incorporação e especialização dos membros de forma semelhante a outros países, como a Inglaterra. O volume de pedidos e a complexidade das decisões, que frequentemente consomem mais de três horas de reunião, como no caso da incorporação de vacinas para COVID-19 em crianças ^7^, inviabilizam a manutenção de um único comitê, cujos membros não são remunerados e ocupam outras funções em seus órgãos de origem.

Quanto à condução política das decisões, em todas as instâncias decisórias há um componente político, uma vez que os decisores representam a sociedade, isso por si só não é errado. O problema é o sequestro político da saúde pública, ao qual nos posicionamos de forma contrária ^8^. Em todas as reuniões que participamos, o plenário da Conitec, em sua maioria, adotou posições corajosas e foi embasado por evidências, como ao aprovar as diretrizes brasileiras para tratamento medicamentoso ambulatorial de pacientes com COVID-19, contraindicando a cloroquina, apesar de várias pressões políticas.

Ainda com relação à COVID-19, houve, sim, “*antecipação à implementação de tecnologias emergentes*”, ao contrário do argumentado no texto. Nos anos de 2021 e 2022, foram elaborados pareceres completos, com modelos econômicos de diversas tecnologias para COVID-19, incluindo todas as vacinas, anticorpos monoclonais, nirmatrelvir/ritonavir e molnupiravir, em diálogo precoce com a Anvisa, permitindo a decisão com suporte de evidências para a emergência de saúde pública, mesmo antes da demanda da indústria. Certamente, o processo de priorização ainda pode ser melhorado, tema que compete às áreas técnicas do Ministério da Saúde, e não à Conitec.

Quanto à heterogeneidade dos pareceres, é um ponto real, mas sem solução imediata. Foram elaboradas diversas diretrizes metodológicas ^9^, publicados editais de fomento para elaboração e atualização de novas diretrizes e métodos inovadores em ATS, ofertados treinamentos e realizadas reuniões para homogeneizar os pareceres. A presença de vários grupos elaboradores de pareceres inevitavelmente causará uma heterogeneidade. Houve muitas críticas no início da Conitec ao pequeno número de grupos elaboradores. Diante da grande demanda, optou-se por aumentar o número de equipes em NATS elaborando pareceres. A decisão estimulou a difusão de conceitos de ATS e o crescimento da Rede Brasileira de Avaliação de Tecnologias em Saúde (Rebrats), incluindo unidades acadêmicas e hospitalares, mas certamente dificultou a homogeneidade da elaboração de pareceres.

Por fim, quanto à inclusão de outros segmentos sociais, acreditamos que os pacientes, maiores interessados no tópico e foco de todo o nosso trabalho, estão inadequadamente representados na Conitec pelo Conselho Nacional de Saúde (CNS), que inclui representantes de pacientes e da indústria farmacêutica. É um desafio conseguir uma representação de pacientes qualificada, que realmente contribua com esse fundamental ponto de vista para a decisão. A participação de pacientes foi ampliada pela criação da experiência do paciente e da consulta pública, mas ainda carece de qualificação e adequada representação. Esforços como o do projeto em construção, ParticipaSUS (https://www.instagram.com/participasus/), tem por objetivo traduzir a complexidade da ATS para pacientes e familiares.

Como todo o projeto de Estado, a avaliação da incorporação de tecnologias em saúde é um assunto complexo, como registrado na *Resolução WHA67.23*^10^, na qual os países-membros da Organização Mundial da Saúde (OMS) reconheceram os enormes desafios na gestão de tecnologias, principalmente em países de baixa e média renda, e a necessidade de fortalecer o vínculo entre avaliação, regulamentação e gestão de tecnologias em saúde.

O debate é benéfico para a sociedade, porém é necessário o conhecimento do contexto histórico e a adequada avaliação dos atores no processo político e das forças incluídas no mesmo. Cabe reconhecer o esforço e a dedicação de toda a equipe da secretaria executiva e dos membros do plenário da Conitec, sem os quais a tomada de decisão estaria restrita a um pequeno grupo de acadêmicos.

## References

[1] Caetano R, Lopes LC, Santos GML, Osorio-de-Castro CGS (2023). Incorporação e uso de medicamentos no Sistema Único de Saúde
mudanças e riscos com os novos atos normativos do Ministério da
Saúde. Cad Saúde Pública.

[2] Leite G (2022). Nova lei de medicamentos para o SUS: pouco deve
mudar.. Outra Saúde.

[3] Ministério da Saúde (2022). Portaria conjunta nº 24, de 7 de dezembro de 2022. Aprova o
Protocolo Clínico e Diretrizes Terapêuticas da Degeneração Macular
Relacionada à Idade.. Diário Oficial da União.

[4] Solomon SD, Lindsley K, Vedula SS, Krzystolik MG, Hawkins BS (2019). Anti-vascular endothelial growth factor for neovascular
age-related macular degeneration. Cochrane Database Syst Rev.

[5] Peres R Ofício nº 324/2021/SEI/GADIP-CG/ANVISA. Processo SEI/MS nº
25000.056762/2020-43..

[6] Matos A (2019). Fiocruz distribui medicamento para transplantados no
SUS.. Agência Fiocruz de Notícias.

[7] Canal da Conitec 13ª Reunião Extraordinária da Conitec - dia 21 12 2022 Medicamentos.
Video: 2:53:29..

[8] Correia LCL, Sette C, Santos M, Magliano CAS, Toscas FS (2022). Brazil's COVID-19 guidelines political hijack of public
health. Lancet.

[9] Comissão Nacional de Incorporação de Tecnologias no Sistema Único de
Saúde Diretrizes metodológicas..

[10] Sixty-Seventh World Health Assembly Health intervention and technology assessment in support of universal
health coverage..

